# An informed regression-based knowledge distillation framework for simultaneous prediction of physical and mechanical properties of thermoset epoxy polymers

**DOI:** 10.1038/s41598-026-61931-7

**Published:** 2026-07-16

**Authors:** B. S. Sindu, Jan Hamaekers

**Affiliations:** 1 Special and Multifunctional Structures Laboratory, CSIR-Structural Engineering Research Centre, Taramani, Chennai, Tamil Nadu 600113 India; 2 Fraunhofer Institute for Algorithms and Scientific Computing SCAI, Schloss Birlinghoven, 53757 Sankt Augustin, Germany

**Keywords:** Engineering, Materials science, Mathematics and computing

## Abstract

Epoxy polymers are widely used due to their multifunctional properties, however their complex 3D molecular structure, multi-component nature, and lack of curated datasets have limited the application of machine learning (ML) for these materials.Existing ML studies are largely restricted to simulation data, specific properties, or narrow constituent ranges. To address these limitations, we developed an Informed Regression-based Knowledge Distillation (R-KD) framework for predicting multiple physical (glass transition temperature, density) and mechanical properties (elastic modulus, tensile strength, flexural strength, adhesive strength) of thermoset epoxy polymers. The model was trained on experimental literature data covering diverse monomer classes (9 resins, 37 hardeners). The best-performing single-task regression model per target property serves as teacher model capturing nonlinear feature-property relationships, while a unified neural network student model learns distilled knowledge across all properties simultaneously. By encoding the target property as an input feature, the student model leverages cross-property correlations. Molecular-level descriptors extracted from SMILES representations using RDKit create a physics-informed model. Comparative analysis demonstrates superior or comparable prediction accuracy over multi-task NN baseline model and conventional ML models. Simultaneous multi-property prediction further improves accuracy through information sharing across correlated properties. The proposed framework enables accelerated design of novel epoxy polymers with tailored properties.

## Introduction

Epoxy polymers are thermoset materials exhibiting multi-functional properties such as high strength, excellent adhesion, effective electrical insulation, low shrinkage, chemical and solvent resistance, etc. They are widely used in several industries like aerospace, marine, automotive, infrastructure, electrical and electronics for specific functional requirement. For instance, adhesive strength, fracture toughness and weight are the major requirements for use in aerospace applications^1^; resistance to moisture, salinity and varied temperature cycles is the major requirement in marine applications; viscosity, low shrinkage and corrosion resistance are the requirements for its application in paint and coatings^1^; insulation resistance is the major requirement for electrical applications^2^; adhesive strength, durability, tensile and fracture properties are the requirements for infrastructural applications^3^. Hence, care should be taken to design epoxy polymers with required functionalities so that they can be used for the intended purposes. The basic constituents of epoxy polymers are resin and hardener. When these two compounds undergo an irreversible reaction, a dense, three dimensional network is formed. However, the functionalities and the properties of epoxy polymers depend upon several factors like the constituent resin and hardener types, their composition, curing conditions and degree of polymerization. The conventional trial-and-error-based experimental investigations limit the development of high performance epoxy polymers with multi-functional characteristics.

ML-based approaches are being widely used for design, optimization and property prediction of homopolymers and copolymers due to the availability of large databanks^4–7^. However, their use in thermoset epoxy polymers is very limited due to the involvement of two or more constituent ingredients, complex three-dimensional structure and lack of large curated datasets. Recently, attempts are being made for composition optimization, property prediction and design of high performance epoxy polymers using ML-based approaches. Results from MD simulations in conjunction with Neural Networks (NNs) were used to optimize the composition of epoxy polymers with performance characteristics like elastic modulus, tensile strength, elongation at break, glass transition temperature ($\textrm{Tg}$)^8^ and self-healing properties^9^. The composition of shape memory epoxy polymers (SMP) was also optimized using NNs using the results from experimental investigations^10^. Unified ML model based on Support Vector Regression (SVR) was proposed to predict the $\textrm{Tg}$ of homo-, hetero- and cross linked epoxy- polymers based on the constituent monomers and its descriptors^11^. ML ensemble model based on Gradient Boosting Regression (GBR) and Kernel Ridge Regression (KRR) was used to predict the $\textrm{Tg}$ of epoxy polymers by correlating the molecular descriptors of resin (16 types) and hardener (19 types) with experimental (94 combinations) DMTA measurements and the most important descriptors affecting the $\textrm{Tg}$ were further identified using Lasso regression^12^. Materials genome approach in conjunction with attention- and gate-augmented graph convolutional networks, multilayer perceptrons and transfer learning was used to identify the gene structures responsible for different properties and to design epoxy polymers with high strength, modulus and toughness^13^. Similarly, ML-based approach was developed to predict the elastic modulus and yield strength of epoxy polymers using the basic structural features of monomers, thereby, enabling feature-based prediction of their properties^14^. ML-based convolutional model was employed for discovery of new thermoset SMPs with high recovery stress from a newly constituted compositional space^15,16^. Physics-Informed Neural Networks (PINN) and Interpretable Machine Learning (XAI) in conjunction with multi-scale modelling were used to predict the thermal conductivity of polyurethane composites^17,18^. The thermomechanical behaviour of shape memory alloy polymers was predicted using Graph Neural Network (GNN) based deep learning framework^19^.

Though several ML approaches have been reported for epoxy polymer property prediction, they are largely restricted to a single target property or a narrow range of constituents, with training data sourced either from computational or experimental investigations. With this in mind, we develop an ML model which can be used to predict multiple physical (glass transition temperature and density) and mechanical (elastic modulus, tensile strength, flexural strength and adhesive strength) properties of two-component, thermoset epoxy polymers merely with the help of its constituent ingredients (type of resin and hardener and its proportion). We develop a Regression-based Knowledge Distillation framework (R-KD) for this purpose in which the best-performing conventional regression modelserve as the teacher model for each target property, and the knowledge learned from these teachers is distilled into a unified student model for property prediction. The constituent resin and hardener are first encoded using a label encoding scheme to numerically represent their chemical identities. These encoded variables are then used together with the relevant process parameters as inputs to the model, allowing it to learn correlations between material composition, processing conditions, and the resulting physical and mechanical properties (cf. “Basic architecture of the model”). We then convert it into an informed ML model^20^ by including information about the intrinsic features of the monomeric constituents extracted from an open source, cheminformatics tool (cf. “Informed R-KD framework”). The data for training the model was collected from several experimental data from literature. We also compared the performance of the model with other conventional single-task regression models as well as a multitask neural network (MT-NN) (baseline model) and found that the proposed R-KD framework achieves comparable or superior prediction accuracy across the majority of target properties, with the most pronounced improvement observed for data-scarce properties (cf. “Improvement due to knowledge distillation” and “Comparison with conventional ML models”). The major advantage of this model is that a single model can be used to predict multiple physical and mechanical properties of epoxy polymers. The model also extracts the benefits from cross-property learning, as training with multiple properties together enables the shared backbone to extract correlated composition-property relationships. This has also been witnessed by improved prediction accuracy while multiple properties are predicted together (cf. “Improvement due to simultaneous prediction of properties”). The model thus paves the path for designing novel epoxy polymers with exceptional properties which would only be possible through laborious experimental trials.

## ML model for prediction of properties

**Fig. 1:**
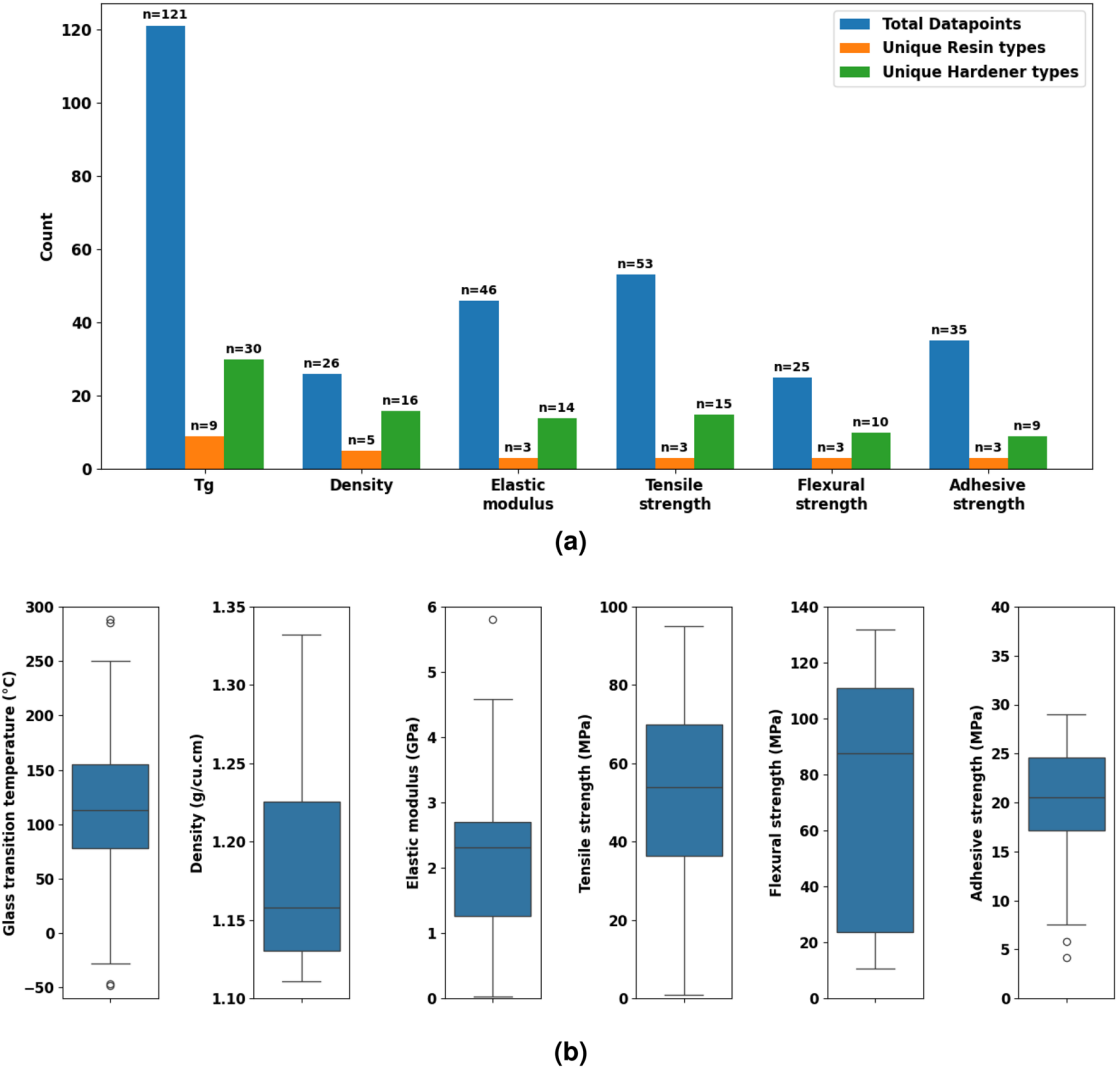
Data collected from literature (used for training the ML model): (**a**) distribution of the dataset showing total number of data points, unique resin types, and unique hardener types available for each target property; (**b**) range of individual physical and mechanical properties.

In order to predict the physical and mechanical properties of epoxy polymers, we develop a Regression-based Knowledge Distillation framework. The data for training the ML model is provided from the experimental results in the literature. A total of 213 data points spread across several properties such as glass transition temperature ($\textrm{Tg}$), density, elastic modulus, tensile strength, flexural strength and adhesive strength for different types of epoxy polymers are collected for this purpose^21–46^. The data spans across diverse resin (9) and hardener (37) classes; two process parameters (stoichiometric ratio between the resin and hardener, curing temperature) and two test parameters (strain rate, test temperature). The distribution of datapoints across different epoxy combinations and the range of individual physical and mechanical properties collected from literature is presented in Fig. 1.

### Basic architecture of the model

**Fig. 2:**
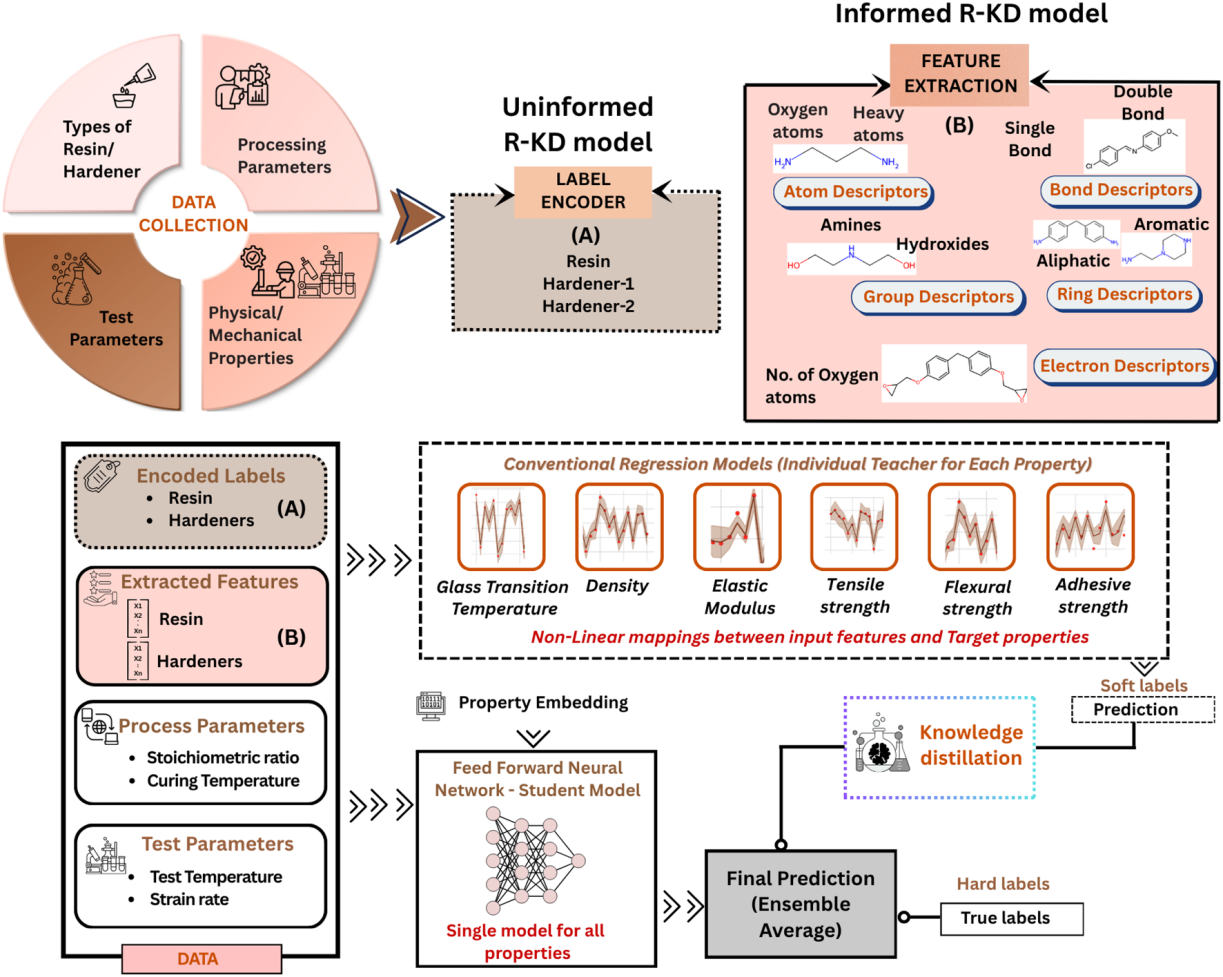
Architecture of the Regression-based Knowledge Distillation (R-KD) framework. Two configurations of the framework are represented: (**A**) uninformed model - resin and hardener molecules are label-encoded, with the encoded labels forming part of the input feature vector; (**B**) informed model - molecular features of the constituent resin and hardeners, extracted using cheminformatics tool, replace the encoded labels as inputs. The two configurations differ only in the input encoding strategy; all other components - process and test parameters, teacher models, knowledge distillation loss, and the student neural network - remain identical.

The basic inputs used for the prediction of epoxy polymer properties using the proposed Regression-based Knowledge Distillation (R-KD) framework are the constituent ingredient (resin and hardener) types, their proportions and other process parameters. Firstly, we convert the categorical data (resin and hardener type) into numerical labels using a label encoder. Then, this encoded information along with the molecular weight of individual monomers, process parameters and test parameters are selected as input features for the model (Fig. 2).

For each target property, an independent regression model is developed and employed as a teacher within the proposed knowledge distillation framework. For training each teacher model, only the subset of the dataset comprising data points associated with the target property is used. The dataset is then normalized to a uniform scale so as to remove bias arising from differences in feature scales. The available data are partitioned into training and testing sets using an 80:20 split. Then, hyperparameter optimization is carried out on training dataset using five-fold cross-validation. A grid search strategy is employed to explore the hyperparameter search spaces of six conventional regression models (as given in Table 1) such as Partial Least Squares (PLS) Regression, Ridge Regression (RR), Random Forest (RF), Gradient Boosting Regression (GBR), k-Nearest Neighbours (kNN), and Gaussian Process Regression (GPR). Model selection is based on maximisation of the ${R^2}$ score on the held-out test set, ensuring that the strongest available model is selected as teacher for each target property. Once the optimal hyperparameter configuration is identified, the best-performing model is retained for final evaluation and knowledge transfer. The trained teacher model is used to generate predictions on the fully normalized input space to produce property-specific soft targets for subsequent knowledge distillation. These conventional regression teacher models thus provide accurate non-linear mappings between the input features and target properties, yielding physically consistent predictive responses that are well suited for guiding the training of a unified student neural network.

**Table 1:** Hyperparameter search space for different regression models.

Model	Hyperparameter	Search space
PLS regression	Number of components	1 to 13
	Tolerance	$10^{-4}$, $10^{-6}$, $10^{-8}$, $10^{-10}$
Ridge regression	$\alpha$	logspace($-10$ to 2)
	Solver	svd, cholesky, lsqr, sparse_cg, sag, saga
Random forest	Maximum depth	[1, 2, 3]
Gradient boosting regression	Learning rate	logspace($-6$ to $-1$)
	Maximum depth	[1, 2, 3]
k-Nearest neighbour	Number of neighbours	1 to 7
	Weights	Uniform, distance
Gaussian process regression	$\alpha$	logspace($-12$ to $-1$)

The student is implemented as a fully connected feed-forward neural network with an input layer, two hidden layers, and an output layer. The input to the network is formed by concatenating the label-encoded resin type, hardener type and molecular weights with a learnable 16-dimensional property embedding, which encodes the target property to be predicted. The property embedding maps each target property index to a continuous vector representation in a 16-dimensional space, allowing the network to capture similarities and correlations between different target properties through the learned embedding geometry. Encoding the target property as an input feature enables a single model to predict all properties simultaneously while sharing a common set of network parameters, thereby allowing cross-property knowledge transfer. The concatenated input is passed sequentially through two hidden layers, where weights and biases are learned through non-linear activation using the rectified linear unit (ReLU) function. Each hidden layer is implemented as a residual block comprising two linear transformations with batch normalisation, ReLU, and dropout (0.1) between them, followed by a skip connection that adds the block input to the output before a final ReLU activation. Through these hidden layers, the model progressively transforms the raw input into a higher-dimensional latent representation that captures coupled constituent–process–property relationships, which are not directly apparent from the original feature space. The final output layer maps this latent representation to a single scalar corresponding to the predicted epoxy polymer property. The architecture of the student network model has been provided in Table 2. The model is trained using PyTorch^47^.

To address the limited availability of experimental data for certain properties, synthetic labels are generated for formulations where measurements are unavailable. For each target property, the selected teacher model predicts the property value for all formulations in the dataset where the property was not experimentally measured, excluding rows that appear in the test set of any property to prevent data leakage. Synthetic labels are clipped to the range of real training observations and diversity-sampled across the prediction range to ensure uniform coverage of the full property space. Synthetic data generation is restricted to properties whose teacher achieves a test $R^2 \ge 0.75$, ensuring that only reliable teacher predictions contribute to synthetic enrichment. The number of synthetic samples per property is capped at the real training count (synthetic ratio $= 1.0$) to prevent synthetic data from dominating the training signal. Properties already at the maximum real sample count receive no synthetic data.

Further, the trained teacher model generates predictions on all enriched training rows (real observed rows plus synthetic rows) to produce property-specific soft targets (predictions generated by the teacher model for each input)). These soft targets encode the teacher’s learned smooth mapping between input data and the target property, providing a richer training signal than the observed hard labels (the actual experimentally measured property values) alone. For synthetic rows, the soft target and the hard label are identical by construction, since both are derived from the same teacher model prediction. During training, a knowledge distillation loss function is employed, defined as a weighted combination of the mean squared error between the student predictions and the teacher soft targets, and the Huber loss with respect to the true experimental values. A weighting factor of $\alpha = 0.35$ is used, assigning higher weight to the experimental observations while retaining guidance from the teacher predictions (the choice was further validated through an ablation study across $\alpha \in \{0.35, 0.50, 0.65\}$, which confirmed that $\alpha = 0.35$ yields the best prediction accuracy). Accordingly, the overall loss function reads as

1 $$\begin{aligned} \mathcal {L}_{\text {KD}} = \alpha \, \text {MSE}(\hat{y}, y_{\text {soft}}) + (1 - \alpha ) \, \text {Huber}(\hat{y}, y_{\text {hard}}). \end{aligned}$$

This distillation strategy enables the student model to benefit from the teacher’s learned feature–property relationships while maintaining direct consistency with experimental observations. Training is carried out using the AdamW optimiser (lr $= 10^{-3}$, weight decay $= 10^{-4}$), mini-batch size of 32, and a maximum of 1000 epochs with early stopping at patience $= 100$. An ensemble of five independently initialised models is trained with different random seeds; final predictions are the mean of all five inverse-transformed outputs, reducing variance arising from random initialisation. The architecture of the R-KD model is given in Table 2.

**Table 2:** Architecture of the student network model.

Hyperparameter	Value
Input dimension	Uninformed model - 26 (10 mol. descriptors + 16 property embeddings)
	Informed model - 66 (50 features + 16 property embeddings)
Hidden layer sizes	2 $\times$ 256 neurons (residual blocks)
Output projection	256 $\rightarrow$ 64 $\rightarrow$ 1
Activation function	ReLU with batch normalisation
Skip connections	Yes (residual blocks)
Dropout rate	0.1
Optimiser	AdamW
Learning rate	$10^{-3}$
Weight decay	$10^{-4}$
Batch size	32
Max epochs	1000
Early stopping patience	100
Ensemble size	5

### Informed R-KD framework

In order to further enhance the predictive capability of the proposed framework as a physics-informed model, intrinsic molecular-level features of the constituent monomers are incorporated. Each resin and hardener monomer is represented by its unique SMILES string^48^, which provides an ASCII-based representation of the molecular structure. From these SMILES strings, atomistic and structural descriptors of the resin and hardener molecules are extracted using the open-source cheminformatics tool RDKit^49^. A total of 25 features which include the following descriptors are extracted from RDKit using a Python script:

- Molecular weight
- Atom descriptors (type and count of atoms including heavy atoms)
- Bond descriptors (single/double/triple)
- Group count (NH/OH, SP3 fractions)
- Ring counts (aromatic/saturated/aliphatic and heterocycles/carbocycles)
- Electron descriptors (No. of hydrogen acceptors/donors, radical/valence electrons)

Together, these five descriptor categories capture the structural connectivity, functional group chemistry, ring topology and electronic characteristics of each monomer molecule, providing a physically meaningful and chemically interpretable representation of the epoxy constituent molecules as input to the framework. In cases where two types of hardeners are present in the formulation, descriptors corresponding to both hardeners are included. In the informed R-KD framework, these molecular descriptors replace the abstract categorical representations used in the uninformed architecture, thereby embedding the resin and hardener molecules directly in their physically meaningful feature space. Since the extracted features span several orders of magnitude, all molecular descriptors are normalized to zero mean and unit variance using StandardScaler to eliminate bias arising from scale differences. A joint feature selection step using mutual information regression selects the 50 most informative features across all target properties simultaneously, ensuring a consistent input space for the shared network backbone.

## Performance of the informed R-KD framework

The performance of the informed R-KD framework in predicting various physical properties (glass transition temperature and density) and mechanical properties (elastic modulus, tensile strength, flexural strength and adhesive strength) of epoxy polymers is evaluated using the dataset shown in Fig. 1. The results of this evaluation are presented and discussed in this section. To assess the statistical reliability of the reported results, the entire pipeline is re-executed across ten independent runs with different random seeds, each comprising a new train/test split, joint feature selection, teacher model selection via grid search cross-validation, synthetic data generation and student NN training. The mean $R^2$ and standard deviation across these ten runs are reported throughout this section. Further, it is to note that given the limited availability of experimental data for several target properties, an 80:20 train/test split yields only 4-10 test samples per property, making the reported $R^2$ score sensitive to the specific composition of the test set. The experimental data compiled from multiple independent literature sources also inherently carry variability arising from differences in testing conditions, specimen preparation and measurement protocols. Both factors contribute to the observed variability across independent runs, and the reported standard deviations reflect data limitations rather than model instability.

### Improvement due to informed configuration

**Fig. 3:**
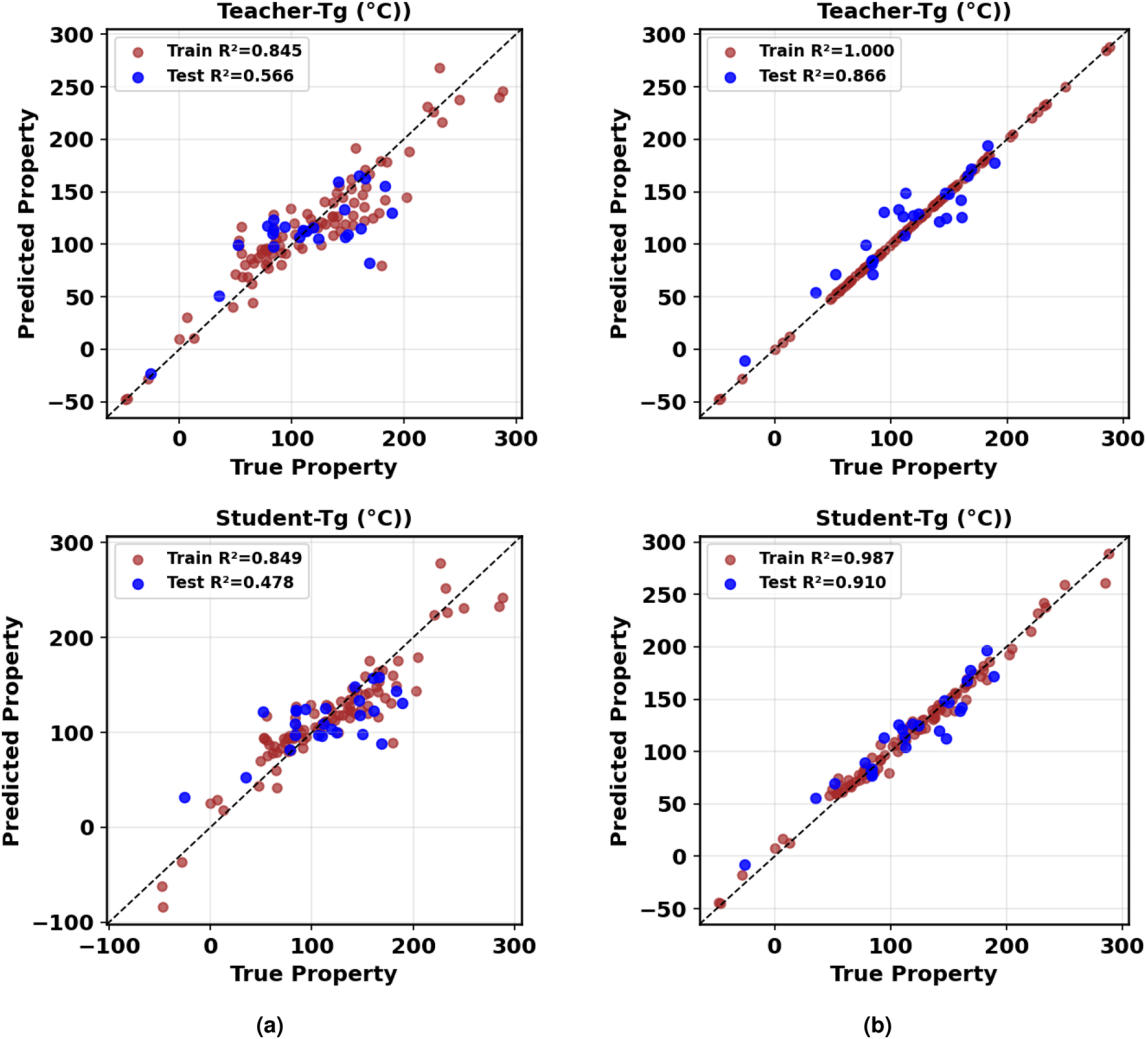
Prediction accuracy of the proposed regression-based knowledge distillation framework (**a**) uninformed configuration, (**b**) informed configuration.

As described in “ML model for prediction of properties”, two configurations of the R-KD framework are considered depending on the input feature representation: (A) the uninformed model, in which the constituent resin and hardener molecules are represented by label-encoded integer indices along with their molecular weights; and (B) the informed model, in which the label-encoded representations are replaced by molecular descriptors extracted from the SMILES strings of the constituent monomers using RDKit. All other components of the framework such as the teacher selection strategy, synthetic data generation, property embedding, student NN architecture, and knowledge distillation loss remain identical across the two configurations, with the only difference being the input encoding strategy. Figure 3 shows the improvement in prediction accuracy of the informed configuration for glass transition temperature. The mean $R^2$ scores across ten repeated random splits for both configurations are presented in Table 3. It can be observed that the informed model achieves superior prediction accuracy for the majority of target properties. The largest improvements are observed for flexural strength ($\Delta R^2 = +0.38$), glass transition temperature ($\Delta R^2 = +0.30$) and adhesive strength ($\Delta R^2 = +0.27$), demonstrating the importance of incorporating chemically meaningful molecular descriptors that capture structure-property relationships not accessible through integer label encodings alone. For tensile strength, both configurations demonstrate comparable performance. Overall, these results confirm that embedding constituent molecules in their physically meaningful feature space substantially enhances the generalisation capability of the student model.

**Table 3:** Prediction accuracy of uninformed and informed R-KD framework.

	Mean $R^2$ ± Std	
Property	Uninformed	Informed
Tg (°C)	$0.52 \pm 0.11$	$\bf 0.82 \pm 0.11$
Flexural strength (MPa)	$0.45 \pm 0.38$	$\bf 0.83 \pm 0.14$
E (GPa)	$0.25 \pm 0.34$	$\bf 0.33 \pm 0.33$
Tensile strength (MPa)	$\bf 0.20 \pm 0.44$	$0.15 \pm 0.60$
Adhesive strength (MPa)	$0.12 \pm 0.84$	$\bf 0.39 \pm 0.52$
Density (g $\text {cm}^{-3}$)	$0.19 \pm 0.60$	$\bf 0.37 \pm 0.49$

Bold values indicate the higher mean R² per property.

### Improvement due to simultaneous prediction of properties

In order to understand the benefit of cross-property knowledge transfer enabled by the multi-task architecture, the informed R-KD framework is tested for predicting each target property independently. The same teacher model, synthetic data generation, knowledge distillation loss is used but with separate model trained for each property. Table 4 presents the comparison of prediction accuracy between the individual and simultaneous R-KD configurations across ten different test/train splits. It is evident that simultaneous multi-property training yields superior prediction accuracy for the majority of properties. The most significant improvements are observed for adhesive strength ($\Delta R^2 = +0.18$), flexural strength ($\Delta R^2 = +0.16$) and elastic modulus ($\Delta R^2 = +0.14$) which have limited experimental data (35, 25 and 46 data points respectively). These results clearly demonstrate that the shared multitask backbone acts as an implicit regulariser wherein gradient updates from data-rich properties regularise the learning of data-scarce properties, enabling the multi-task R-KD framework to generalise well beyond what is achievable from limited single-property training data alone. For glass transition temperature, the individual model performs marginally better ($\Delta R^2 = -0.06$), consistent with the expectation that data-rich properties (121 data points) benefit less from cross-property regularisation and may experience mild interference from the shared backbone.

**Table 4:** Prediction accuracy of Informed R-KD framework during simultaneous prediction of properties.

	Mean $R^2$ ± std	
Property	Individual KD	Multitask KD
Tg (°C)	$\bf 0.87 \pm 0.06$	$0.82 \pm 0.11$
Flexural strength (MPa)	$0.67 \pm 0.22$	$\bf 0.83 \pm 0.14$
E (GPa)	$0.19 \pm 0.41$	$\bf 0.33 \pm 0.33$
Tensile strength (MPa)	$0.11 \pm 0.55$	$\bf 0.15 \pm 0.60$
Adhesive strength (MPa)	$0.22 \pm 0.51$	$\bf 0.39 \pm 0.52$
Density (g $\textrm{cm}^{-3}$)	$\bf 0.38 \pm 0.70$	$0.37 \pm 0.49$

Bold values indicate the higher mean R² per property.

### Improvement due to knowledge distillation

The prediction accuracy of informed multi-task R-KD model is also compared with that of informed multi-task NN model (MT-NN) which is the baseline model with similar architecture (excluding synthetic data augmentation and knowledge distillation block). Table 5 presents the prediction accuracy of both the configurations across 10 different test/train splits. It can be observed that the R-KD framework consistently outperforms the MT-NN baseline for all six target properties, demonstrating the contribution of the knowledge distillation mechanism to the overall prediction accuracy. The most substantial improvements are observed for adhesive strength ($\Delta R^2 = +0.54$) and tensile strength ($\Delta R^2 = +0.24$), where the MT-NN completely fails to generalise across repeated runs while the R-KD framework maintains consistently positive prediction accuracy. Improvements are also observed for the remaining properties, confirming that knowledge distillation provides a consistent improvement over the multitask NN trained without soft targets and synthetic data.

**Table 5:** Prediction accuracy of informed multi-task KD framework in comparison with MT-NN baseline model.

	Mean $R^2$ ± std	
Property	MT-NN	R-KD
Tg (°C)	$0.75\pm 0.12$	$\bf {0.82\pm 0.11}$
Flexural strength (MPa)	$0.78\pm 0.21$	$\bf {0.83\pm 0.14}$
E (GPa)	$0.23\pm 0.34$	$\bf {0.33\pm 0.33}$
Tensile strength (MPa)	$-0.09\pm 0.83$	$\bf {0.15\pm 0.60}$
Adhesive strength (MPa)	$-0.15\pm 1.07$	$\bf {0.39\pm 0.52}$
Density (g $\textrm{cm}^{-3}$)	$0.21\pm 0.67$	$\bf {0.37\pm 0.49}$

Bold values indicate the higher mean R² per property.

### Comparison with conventional ML models

The performance of the informed R-KD framework is compared with that of conventional single-task regression models (PLS, Ridge, RF, GBR, kNN and GPR) as described in “Basic architecture of the model” across 10 different test/train splits. Table 6 presents the prediction accuracy of all the models. It can be observed that the informed R-KD framework achieves comparable or superior prediction accuracy to the best-performing conventional single-task model for the majority of the properties. The most pronounced improvements are observed for adhesive strength and tensile strength, where the majority of conventional models yield negative mean $R^2$ scores while the R-KD framework maintains positive prediction accuracy, demonstrating the benefit of cross-property knowledge transfer for data-scarce properties. For properties with relatively more data, such as glass transition temperature and flexural strength, the R-KD framework achieves comparable or superior accuracy to the best conventional model, confirming that simultaneous multi-property training does not compromise accuracy for better-represented properties. Furthermore, the R-KD framework offers a significant practical advantage: a single unified model simultaneously predicts all target properties, eliminating the need for independently trained and maintained single-task models.

**Table 6:** Prediction accuracy of R-KD framework in comparison with conventional models.

	Mean $R^2$ ± std						
Property	PLS	Ridge	RF	GBR	kNN	GPR	R-KD
Tg (°C)	$0.45\pm 0.22$	$0.52\pm 0.16$	$0.50\pm 0.15$	${\bf 0.82\pm 0.07}$	$0.79\pm 0.13$	$0.74\pm 0.13$	${\bf 0.82\pm 0.11}$
Flexural strength (MPa)	$0.49\pm 0.37$	$0.45\pm 0.18$	$0.68\pm 0.19$	$0.20\pm 0.70$	$0.65\pm 0.27$	$0.70\pm 0.24$	${\bf 0.83\pm 0.14}$
E (GPa)	$0.20\pm 0.53$	$0.17\pm 0.47$	$0.23\pm 0.46$	$0.08\pm 0.44$	$0.24\pm 0.24$	$0.00\pm 0.43$	${\bf 0.33\pm 0.33}$
Tensile strength (MPa)	$-0.32\pm 0.68$	$-0.23\pm 0.66$	$0.06\pm 0.63$	$-0.41\pm 0.92$	$-0.03\pm 0.63$	$-0.14\pm 0.67$	${\bf 0.15\pm 0.60}$
Adhesive strength (MPa)	$-0.57\pm 2.00$	$-0.18\pm 1.15$	$0.07\pm 0.60$	$-0.24\pm 1.27$	$-0.20\pm 1.52$	$0.06\pm 0.91$	${\bf 0.39\pm 0.52}$
Density (g $\textrm{cm}^{-3}$)	$0.38\pm 0.42$	$0.37\pm 0.61$	$0.38\pm 0.40$	$0.21\pm 0.48$	${\bf 0.41\pm 0.47}$	$0.12\pm 0.88$	$0.37\pm 0.49$

Bold values indicate the best mean R² per property

## Conclusions

In this study, an attempt has been made to develop an informed Regression-based Knowledge Distillation (R-KD) framework for prediction of various physical (density and glass transition temperature) and mechanical properties (flexural strength, elastic modulus, tensile strength and adhesive strength) of thermoset epoxy polymers. Data from literature pertaining to experimental investigations on epoxy polymers covering wide range of resin/hardener classes and different physical and mechanical properties have been used for training the model. Following are some of the salient features of the proposed R–KD framework:

- A hybrid teacher–student architecture in which the best-performing single-task regression model per target property serves as teacher to guide a multi-task neural network student through knowledge distillation.
- Knowledge distillation enables the student network to inherit the generalization capability and regularization behavior of teacher models while maintaining direct consistency with experimental observations through a weighted combination of teacher-guided soft targets and experimental hard labels.
- The framework explicitly incorporates characteristic descriptors of constituent resin and hardener molecules, allowing chemically meaningful learning of composition–property relationships. Since the model operates on molecular descriptors rather than categorical constituent labels, any new polymer system can be evaluated as long as its molecular descriptors can be extracted
- A learnable property embedding conditions the shared network backbone on the target property, facilitating cross-property knowledge transfer through the learned embedding geometry.
- A single distilled student model is capable of predicting multiple physical and mechanical properties simultaneously, eliminating the need for separate models for individual targets, offering a computationally efficient and scalable alternative as larger datasets become available.
- The R-KD framework demonstrates improved prediction accuracy compared to conventional standalone machine learning models and the multitask NN baseline, particularly in data-scarce regimes, confirming the effectiveness of the proposed knowledge distillation strategy for epoxy polymer property prediction.
- The proposed approach is well suited for high-throughput screening and design of epoxy systems, where candidate resin-hardener formulations can be rapidly evaluated for multiple target properties simultaneously using only their constituent SMILES representations and process parameters as inputs, enabling accelerated exploration of composition-processing-property relationships and discovery of epoxy systems with tailored properties.

This model thus paves the way for design of epoxy polymers with desired physical and mechanical properties thereby leading towards sustainable development. However, since the experimental data used in this study were compiled from multiple independent literature sources, they inherently carry variability arising from differences in testing conditions, specimen geometry and measurement protocols across studies. There remains a broader need for generating large, systematically curated datasets to enable more robust and reliable machine learning model development in this field. Future work will focus on experimental validation of predicted formulations, extension to other thermoset polymer systems, and incorporation of physics-informed neural network approaches. Additionally, quantitative sensitivity analysis of key molecular descriptors and input parameters is envisaged to provide deeper insight into composition-property relationships and further strengthen model interpretability.

## Supplementary Information

Supplementary Information 1.

Supplementary Information 2.

## Data Availability

The complete dataset used for training and testing the proposed R-KD framework has been provided as part of the supplementary material.
