# Supplementary material for: An informed regression-based knowledge distillation framework for simultaneous prediction of physical and mechanical properties of thermoset epoxy polymers
Source: Sci Rep. 2026 Jul 16;16:22411. doi: 10.1038/s41598-026-61931-7 (PMC13376364; doi:10.1038/s41598-026-61931-7)
Supplement: Supplementary file 1 — Supplementary Information 1. [file 41598_2026_61931_MOESM1_ESM.pdf]

**Supplementary file**

**Table 1A Resin types used in this study**

| S.No. | Resin Name | SMILES |
|---|---|---|
| 1. | Diglycidyl ether of bisphenol A (DGEBA) | CC(C)(c1ccc(cc1)OCC2CO2)c3ccc(cc3)OCC4CO4 |
| 2. | Diglycidyl ether of ethylene glycol (DGEE) | C(COCC1CO1)OCC2CO2 |
| 3. | Diglycidyl ether of 1,4-butanediol (DGEBD) | C1C(O1)COCCCCOCC2CO2 |
| 4. | Diglycidyl ether of glycerol (DGEB) | C1C(O1)COCC(CO)OCC1CO1 |
| 5. | Diglycidyl resorcinol ether (DGEPM) | c3cc(OCC1CO1)cc(OCC2CO2)c3 |
| 6. | Hydroquinone diglycidyl ether | c2cc(OCC1CO1)ccc2OCC3CO3 |
| 7. | Triglycidyl amino phenol (TGAP) | c3cc(N(CC1CO1)CC2CO2)ccc3OCC4CO4 |
| 8. | Diglycidyl ether of bisphenol F (DGEBF) | c1cc(ccc1Cc2ccc(cc2)OCC3CO3)OCC4CO4 |
| 9. | Tetraglycidyl methylenedianiline (TGDDM) | c1cc(ccc1Cc2ccc(cc2)N(CC3CO3)CC4CO4)N(CC5CO5)CC6CO6 |

**Table 2A Hardener types used in this study**

| S.No. | Hardener Name | SMILES |
|---|---|---|
| 1. | Dodecane diamine (DA12) | C(CCCCCCN)CCCCCN |
| 2. | Hexane diamine (DA6) | C(CCCN)CCN |
| 3. | Butane diamine (DA4) | C(CCN)CN |
| 4. | Diaminopropane (DAP) | C(CN)CN |
| 5. | Ethylenediamine (EDA) | C(CN)N |
| 6. | Triethylenetetramine (TETA) | C(CNCCNCCN)N |

| | | |
|---|---|---|
| 7. | Tetraethylenepentamine (TEPA) | C(CNCCNCCNCCN)N |
| 8. | Diethanolamine (DEA) | C(CO)NCCO |
| 9. | Trimethylene glycol di-p-aminobenzoate (TMAB) | C(OCCCOC(=O)C1=CC=C(N)C=C1)(=O)C2=CC=C(N)C=C2 |
| 10. | 1,3-Bisaminomethylcyclohexane (13BAC) | C1CC(CC(C1)CN)CN |
| 11. | 4,4′-methylenebis(cyclohexylamine) (PACM) | C1CC(CCC1CC2CCC(CC2)N)N |
| 12. | Piperidine (PIP) | C1CCNCC1 |
| 13. | 2,2′-Bis(4-(4-aminophenoxyl)phenyl)propane (BAPP) | CC(C)(c1ccc(cc1)Oc2ccc(cc2)N)c3ccc(cc3)Oc4ccc(cc4)N |
| 14. | Jeffamine EDR-148 | CC(COCC(C)OCC(C)OCC(C)N)N |
| 15. | 1-(2-Aminoethyl)piperazine (AEP) | CC(N)N1CCNCC1 |
| 16. | Isophorone diamine (IPD) | CC1(CC(CC(C1)(C)CN)N)C |
| 17. | Methylhexahydrophthalic anhydride (MTHPA) | CC12CCCCC1C(=O)OC2=O |
| 18. | Methylnadic anhydride (MNA) | CC1=CC2CC1C3C2C(=O)OC3=O |
| 19. | 3,5,5-Trimethylcyclohexylamine (TMCA) | CC1CC(CC(C1)(C)C)N |
| 20. | Jeffamine T-403 | CCC(COCC(C)C)(COCC(C)C)COCC(C)N |
| 21. | Diethyltoluenediamine (DETDA) | CCc1cc(c(c(c1N)C)N)CC |
| 22. | 4,4′-Methylenebis(3-chloro-2,6-diethylaniline) (MCDEA) | CCc1cc(c(c(c1N)CC)Cl)Cc2cc(c(c(c2Cl)CC)N)CC |
| 23. | Tris(dimethylaminomethyl) phenol (DMP) | CN(C)Cc1cc(c(c(c1)CN(C)C)O)CN(C)C |
| 24. | Poly(3-aminopropylmethylsilane) (PAMS) | CO[Si](CCCN)(OC)OC |
| 25. | 4,4′-Methylenebis(2,6-diethylaniline) (MDEA) | Cc1cc(cc(c1N)C)Cc2cc(c(c(c2)C)N)C |

| | | |
|---|---|---|
| 26. | 4,4′-Diamino-3,3′-dimethyldicyclohexylmethane (3DCM) | Cc2cc(Cc1ccc(N)c(C)c1)ccc2N |
| 27. | Diethylenetriamine (DETA) | NCCNCCN |
| 28. | 2,6-Diaminopyridine | c1cc([nH]c(=N)c1)N |
| 29. | M-Phenylenediamine (MPDA) | c1cc(cc(c1)N)N |
| 30. | 3,3′-Diaminodiphenyl sulfone (33DDS) | c1cc(cc(c1)S(=O)(=O)c2cccc(c2)N)N |
| 31. | 4,4′-Diaminodiphenyl methane (DDM) | c1cc(ccc1Cc2ccc(cc2)N)N |
| 32. | Bis(4-(4-aminophenoxy)phenyl) sulfone (BAPS) | c1cc(ccc1N)Oc2ccc(cc2)S(=O)(=O)c3ccc(cc3)Oc4ccc(cc4)N |
| 33. | 4,4′-Diaminodiphenyl sulfone (44DDS) | c1cc(ccc1N)S(=O)(=O)c2ccc(cc2)N |
| 34. | Aniline | c1ccc(cc1)N |
| 35. | Phthalic anhydride (PA) | c1ccc2c(c1)C(=O)OC2=O |
| 36. | Poly(methyl methacrylate) (PMMA) | CCC(C)(C(=O)OC)CC(C)(C(=O)OC)CC(C)(C(=O)OC)CC(C)(C(=O)OC)CC(C)(C(=O)OC)C |
| 37. | 4,4′-Methylenebis(2-isopropyl-6-methylaniline) (MPMA) | Cc1cc(cc(c1N)C(C)C)Cc2cc(c(c(c2)C(C)C)N)C |
